# Correlated clusters of closed reaction centers during induction of intact cells of photosynthetic bacteria

**DOI:** 10.1038/s41598-020-70966-3

**Published:** 2020-08-19

**Authors:** Péter Maróti, István A. Kovács, Mariann Kis, James L. Smart, Ferenc Iglói

**Affiliations:** 1 Department of Medical Physics and Informatics, Szeged University, Rerrich Béla tér 1., 6720 Szeged, Hungary; 2 Department of Physics and Astronomy, Northwestern University, Evanston, IL 60208-3112 USA; 3 Wigner Research Centre for Physics, Institute for Solid State Physics and Optics, P.O. Box 49, 1525 Budapest, Hungary; 4 Department of Network and Data Science, Central European University, Budapest, 1051 Hungary; 5 Department of Biological Sciences, University of Tennessee at Martin, Martin, TN 38238 USA; 6 Institute of Theoretical Physics, Szeged University, 6720 Szeged, Hungary

**Keywords:** Biophysics, Plant sciences, Mathematics and computing, Optics and photonics

## Abstract

Antenna systems serve to absorb light and to transmit excitation energy to the reaction center (RC) in photosynthetic organisms. As the emitted (bacterio)chlorophyll fluorescence competes with the photochemical utilization of the excitation, the measured fluorescence yield is informed by the migration of the excitation in the antenna. In this work, the fluorescence yield concomitant with the oxidized dimer (P^+^) of the RC were measured during light excitation (induction) and relaxation (in the dark) for whole cells of photosynthetic bacterium *Rhodobacter sphaeroides* lacking cytochrome *c*_2_ as natural electron donor to P^+^ (mutant *cycA*). The relationship between the fluorescence yield and P^+^ (fraction of closed RC) showed deviations from the standard Joliot–Lavergne–Trissl model: (1) the hyperbola is not symmetric and (2) exhibits hysteresis. These phenomena originate from the difference between the delays of fluorescence relative to P^+^ kinetics during induction and relaxation, and in structural terms from the non-random distribution of the closed RCs during induction. The experimental findings are supported by Monte Carlo simulations and by results from statistical physics based on random walk approximations of the excitation in the antenna. The applied mathematical treatment demonstrates the generalization of the standard theory and sets the stage for a more adequate description of the long-debated kinetics of fluorescence and of the delicate control and balance between efficient light harvest and photoprotection in photosynthetic organisms.

## Introduction

Photosynthesis is responsible for the genesis, development and regulation of vast majority forms of life on the Earth by using the ultimate free energy source of the sun. The conversion of (sun)light to chemical energy is initiated by the absorption of the photons in the closely packed network of protein–pigment complexes (antenna) followed by funneling of the excitation energy (exciton) to a specially organized (B)Chl dimer (P) in the reaction centers (RC)^1^. Here an electron is stripped from P (P$\rightarrow$P^+^) converting the energy of the exciton into chemical (redox) energy of P/P^+^. The electron is transferred via the primary quinone acceptor Q_A_ to the secondary quinone acceptor Q_B_ producing a series of transient charge separated states (P^+^Q^–^). While Q_A_ can accept one electron only, Q_B_ performs two-electron chemistry: by binding two protons and forming reduced quinone QH_2_, it is exchanged for an oxidized quinone from the quinone pool in the membrane^2–4^.

To describe the functional cooperation of the antenna pigments in light collection, the loose concept of the photosynthetic unit (PSU) was introduced^5^. According to the present knowledge, the structure of the PSU of photosynthetic bacteria can be identified as the core complex including the photochemical RC and the closely attached light-harvesting (core) antenna (LH1, B870 in *Rhodobacter (Rba.) sphaeroides*) together (if exists) with the peripheral antenna (LH2, B800-850 complex in *Rba. sphaeroides*) loosely arranged in the photosynthetic membrane (fluid–mosaic-membrane model)^6^. The energy of light harvested by LH2 is transferred to LH1, which directs these excitations (excitons) to an open RC. Here the migration of the exciton in the antenna is terminated, as it is trapped by the RC, which then becomes closed (photochemically incompetent). Another exciton visiting the closed RC can be redirected to an open RC. The peripheral LH2 controls the exciton transfer out of the PSU and acts as a sort of insulator between the PSUs (it can decrease the rate of the inter-unit transfer of the excitons). The exciton is able to visit several PSUs during its lifetime. The search for utilization of the exciton by photochemistry (charge separation) competes with loss by fluorescence emission. This competition is manifested in an inverse relation between energy trapping in RCs and fluorescence yield of the light harvesting bacteriochlorophylls, first recognized in photosynthetic purple bacterium by Vredenberg and Duysens in 1963^7^. This discovery initiated a wealth of studies on the ways and kinetics of the RC occupation by the excitons and its correlation with the change of the BChl fluorescence yield. As the excitions can visit several PSUs, the first studies assumed free diffusion of excitons over very large region (“lake model”^8–10^). However, limitations due to the structural organization of the antenna and to kinetic constraints restricted the number of visits to a few PSUs (“connected units model”^11,12^).

There are two distinct methods to describe the migration of the excitons and the closure of the RCs. The first assumes homogeneous distribution of the reactants combined with small set of reaction rate constants^12,13^. This simplified treatment has the advantage of digestible interpretation of the experimental results by solution of set of ordinary differential equations. The second is a more accurate treatment of the exciton diffusion using either the master equation approach^9,14,15^, Monte Carlo (MC) calculations^19^, Markov chain^16^, or fractional-dimension diffusion model^17^. The disadvantage of this method is the partial loss of the possibility of straightforward comparison of the outcomes with the experimental results and of direct correspondence of the parameters with the measurable quantities.

The very recent membrane scale models of light harvesting are of great interest and significance, but they do not analyze and forecast the behavior of induction and relaxation of fluorescence^18^. In the present work, the problem of excitonic connectivity in bacterial antenna system is revisited, addressing both experimental and theoretical aspects. The experimental section demonstrates remarkable findings on fluorescence and absorption change kinetics both under continuous excitation (induction) and subsequently in the dark (relaxation). We observed (1) the enhancement of the absorption cross section of the open RC when its neighbors were closed and (2) a clustering of closed RCs during induction that failed during relaxation. The theoretical section aims to provide a comprehensive toolbox to handle the exciton migration within the organized antenna, terminating in capture by an open RC (photochemical utilization) or waste by fluorescence emission. This theory relies on a random walk of the excitons on a two-dimensional square lattice and permits temporal evaluation of the state of the RC (open vs. closed) and of the fluorescence both upon continuous excitation and in the dark. The method sets the stage to understand the observed complexity of the fluorescence induction and relaxation kinetics in purple bacteria^20,21^ and in PSII of higher plants^22^. A direct comparison is made with the results from the homogeneous kinetic (Joliot) model. In the future, the theoretical treatment can be extended for more accurate description of the exciton diffusion in various types of and more realistic models of antenna and RC organization supported by recent electron and atomic force microscopy^23^.

## Results

### Experimental results and the Joliot model

#### Kinetics of fluorescence and absorption change

**Figure 1:**
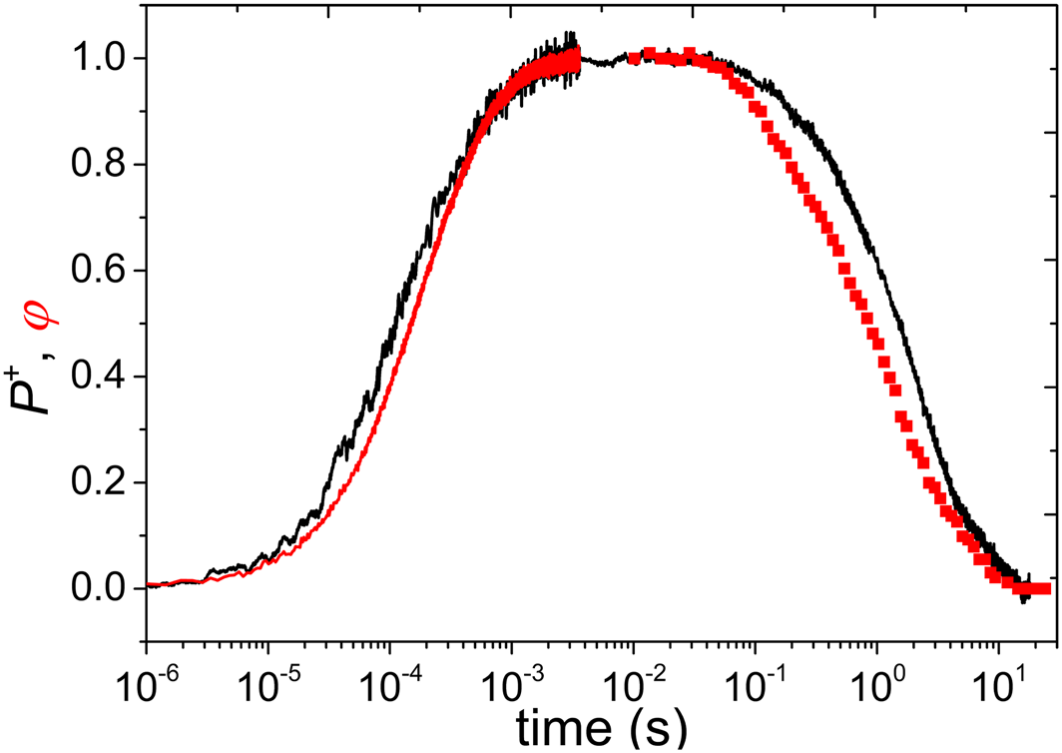
Kinetics of fluorescence yield ($\varphi$) and absorption changes of oxidized dimer (P^+^) during induction and relaxation of whole cells of cytochrome *c*_2_ less mutant of purple photosynthetic bacterium *Rba. sphaeroides*. Both $\varphi$ and P^+^ are normalized to their maximum values.

One of the most convenient methods to study the excitonic coupling among the PSUs is the measurement of the kinetics of the induction and subsequent relaxation of the yield of fluorescence emitted by the BChl antenna. The rise in fluorescence was detected upon laser diode excitation and the decay was monitored by a series of short laser diode probing flashes. A typical experiment is shown in Fig. 1 on whole cells of the cyt *c*_2_ less mutant of photosynthetic purple bacterium *Rba. sphaeroides* (*cycA* strain). As the variant lacks natural electron donors to P^+^, single turnover of the RC is assured upon excitation whose duration is less than the P^+^Q_A_^–^$\rightarrow$PQ_A_ charge recombination time ($\sim$100 ms). The fluorescence (induction) followed the PQ_A_ $\Rightarrow$ P^+^Q_A_^–^ photochemistry with rise time inversely proportional to the exciting light intensity. A rise time in the submillisecond time range was selected to avoid complications with the appearance of short lived triplet quenchers and with the charge recombination on the microsecond and 100 ms time scales, respectively. The applied light intensity was able to saturate the fluorescence within 5 ms. The saturated high fluorescence state was a long lived state indicated by the slow relaxation of the fluorescence in the dark ($\sim$ 1 s) in accordance with the re-reduction of P^+^ by the P^+^Q_B_^–^$\rightarrow$PQ_B_ charge recombination.

To monitor the time course of P^+^, the kinetics of absorption change at 790 nm were measured. This signal is attributable to an electrochromic shift of the absorption band of the BChl monomers in the RC that is induced by the dimer. The conditions were the same in fluorescence and absorption experiments both during induction and in relaxation. While the rise of the fluorescence was slower with respect to absorption change in the induction, the decay of fluorescence was faster than that of the absorption in the relaxation. As both signals were normalized to their full amplitude, we conclude that the fluorescence was always below the absorption. The observed difference in the kinetics reflects the excitonic connectivity of the PSU. Additionally, the difference between the kinetic traces of fluorescence and absorption change is larger during relaxation than during induction.

#### Exciton wandering model: a reminder

Here we recapitulate the essence of an exciton wandering model, which aims to describe the particular kinetics of rise and decay of fluorescence and oxidation of P and re-reduction of P^+^. We are interested in the time-dependence of the fraction of open and closed RCs, *P*(*t*) and ${P^+}(t)$, respectively and their relation to the yield of the fluorescence $\varphi$.

Within this model each RC is identified as the site within a lattice, $i=1,2,\ldots ,N$, which can be in two different states,  for an open RC with a value $\sigma_i=0$ and  for a closed RC with $\sigma_i=1$. The fraction of closed RCs: $P^+=\lim_{N \rightarrow \infty }\frac{1}{N}\sum_{i=1}^N \sigma_i=\langle \sigma \rangle$ is the order-parameter. At the starting point of the induction all RCs are open, thus $P^+(0) = 0$ followed by continuous closure of the RCs. For long enough time, all the RCs become closed, thus $P^+ = 1$. During relaxation, we start from a fully-closed state, and after a sufficient period of time, a fraction of the RCs $(1-P^+)$ will spontaneously reopen. Using an appropriate weak probing light beam, the dynamics of the system can be studied in this case, too.

In the direct process, when an incoming exciton (denoted by  ) hits an open RC, say at $i_1$, the RC will become closed, which is represented graphically in the first line of Eq. (1). If, however, the exciton hits a closed RC, two processes can take place. With probability *p*, the exciton visits a neighbouring RC, or with probability $(1-p)$ the energy of the exciton is dissipated by emission of a fluorescence quantum, $\mathbf{pF}$, see the second line of Eq. (1).1Depending on the lifetime of the exciton it can jump to a new site, say to $i_2$, and the same state-dependent processes can take place, as represented in Eq. (1). This is repeated for more jumps, say on the route of closed sites $i_1 \curvearrowright i_2 \curvearrowright i_3 \curvearrowright \ldots \curvearrowright i_k$, and the processes at $i_k$ are the same as in Eq. (1). Thus, the experimentally measured fluorescence yield is given as the sum of the contributions of the different processes:

2 $$\begin{aligned} \varphi =\sum_{k=1}^n \varphi_k;\quad \varphi_k=(1-p)p^k G_k,\; k<n, \end{aligned}$$

and at the last step $\varphi_n=p^n G_n$, where *n* characterises the life-time of the exciton and $G_k$ is the probability, that the exciton visited at each step a closed RC. In a diagrammatic way $\varphi_k,\; k<n$ is represented as:3

#### Deviations from the Joliot model

In the *Joliot model* the following approximations are used: (1) the life-time of the exciton is unlimited, and (2) the exciton can visit any sites of the lattice with the same probability. From the second condition, which corresponds to the (standard) mean-field treatment of the problem, the multisite correlations are expressed as $G_k=(P^+)^k$. Then, due to the first condition the fluorescence yield in Eq. (2) assumes the form of a geometric series from which a hyperbolic (and not a linear) relation follows:

4 $$\begin{aligned} \frac{1}{\varphi (t)}=\frac{1}{(1-p)} \frac{1}{P^+}-\frac{p}{1-p}. \end{aligned}$$

**Figure 2:**
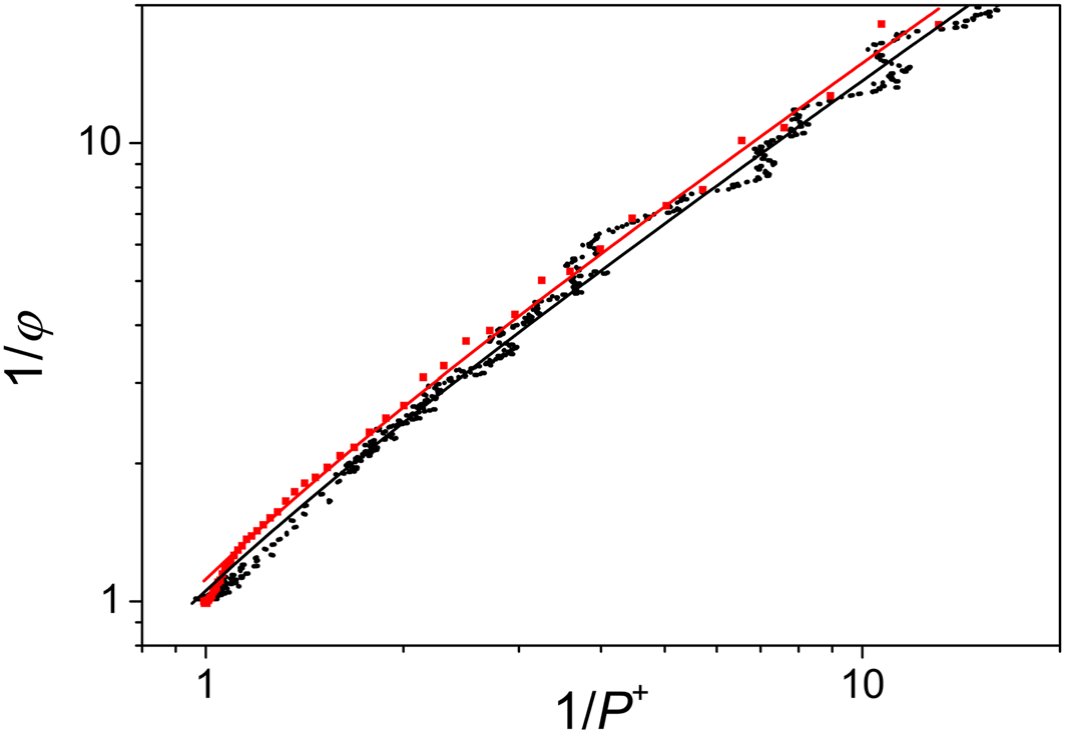
Double reciprocal representations of the fluorescence yield ($\varphi$) and oxidized dimer (P^+^) during induction and relaxation. The plots demonstrate the deviation from the Joliot model that predicts straight lines (curved in the logarithmic scale).

The validity of this expression can be checked by simultaneous measurement of fluorescence and *P*^+^ from absorption change. In double reciprocal representation, systematic deviation from the straight line can be observed both during induction and during relaxation (Fig. 2). The divergence is large in the vicinity of the borders (0 and 1).

The dynamics of closing the open RCs follows from the fact that all incoming photons that are not emitted through fluorescence will reduce the number of open RCs, thus the time-dependence of *P* (and similarly of $P^+$) follows the rule:

5 $$\begin{aligned} -\frac{\text {d} P}{\text {d} t}=\frac{\text {d} {P^+}}{\text {d} t}=k_I(1-\varphi ), \end{aligned}$$

where $k_I$ is the photochemical rate constant (time-scaling factor). Integrating this equation we obtain for the complementary area of the fluorescence, $C(t)=\int_0^t(1-\varphi (t')) {\text {d}} t'$, which is defined as the area that is above the fluorescence rise during induction and below the fluorescence decay during relaxation, and which is related to the fluorescence yield as:

6 $$\begin{aligned} \frac{1}{\varphi (t)}=\frac{1}{k_I (1-p)} \frac{1}{C(t)}-\frac{p}{1-p}. \end{aligned}$$

**Figure 3:**
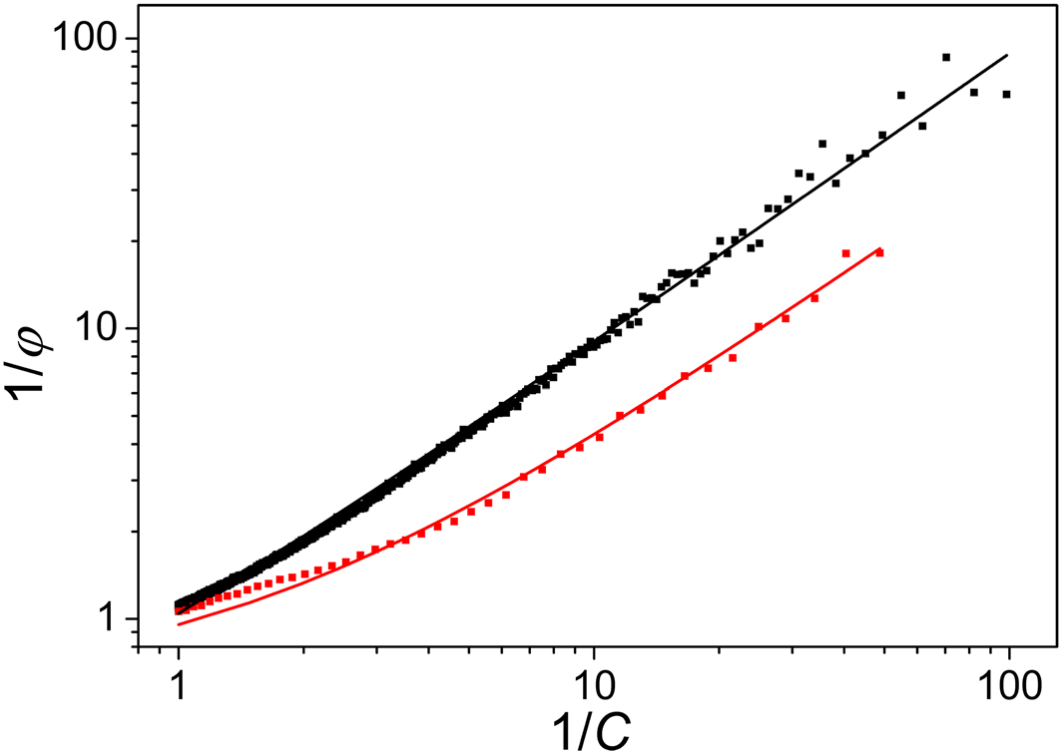
Double reciprocal plots of the fluorescence yield ($\varphi$) and complementary area (*C*) above the fluorescence rise (induction) or below the fluorescence drop (relaxation), respectively. Systematic deviations can be observed from straight lines predicted by the Joliot model. Note, that the straight lines are curved in logarithmic scales.

Equation (6) predicts that the fluorescence data in double-reciprocal representation of $\varphi (t)$ versus *C*(*t*) should be linear. Although the plots in Fig. 3 are close to straight lines, the measured data do not scatter randomly around the straight line: the deviations are systematic. They are smaller during induction and larger during relaxation of the fluorescence.

#### Hysteresis of the $\varphi$ versus P^+^ relationship

**Figure 4:**
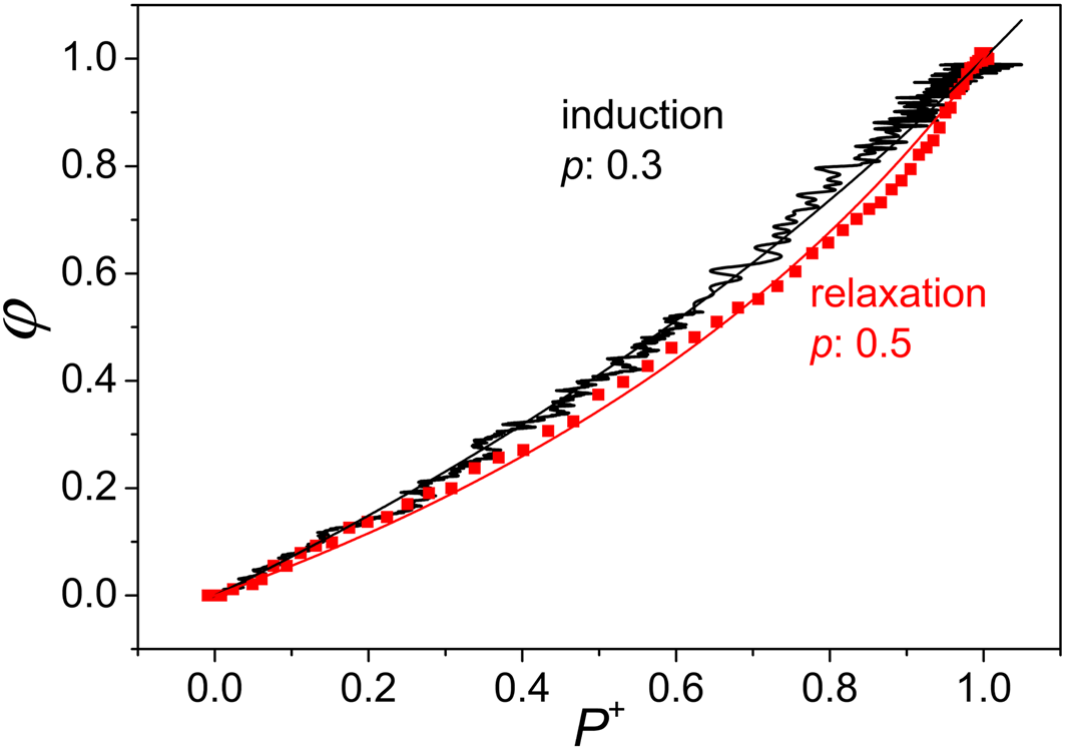
Fluorescence yield ($\varphi$) as a function of closure of the reaction centers (P^+^) during induction and relaxation phases obtained by comparison of the kinetics of fluorescence with those of the oxidised dimer by elimination of the time. The cells were harvested in the late stationary phase of their growth (3 days after inoculation). The measured points were formally approximated by curves derived from the Joliot model with different *p* values indicated. The straight line corresponds to $p=0$, i.e. no connection between the PSUs. The hysteresis (the difference between induction and relaxation) is relatively modest.

As we saw above, the rise in fluorescence is slower than P^+^ upon illumination (induction), and the decay is faster than P^+^ in the dark (relaxation). Furthermore, these two deviations are different: the delay in fluorescence rise relative to P^+^ during induction is smaller than the increase in decay that we observed during relaxation in the dark. This difference results in a hysteresis observable in the plot of $\varphi$ versus P^+^ that can be obtained from the kinetic data after elimination of the time variable (Fig. 4).

**Figure 5:**
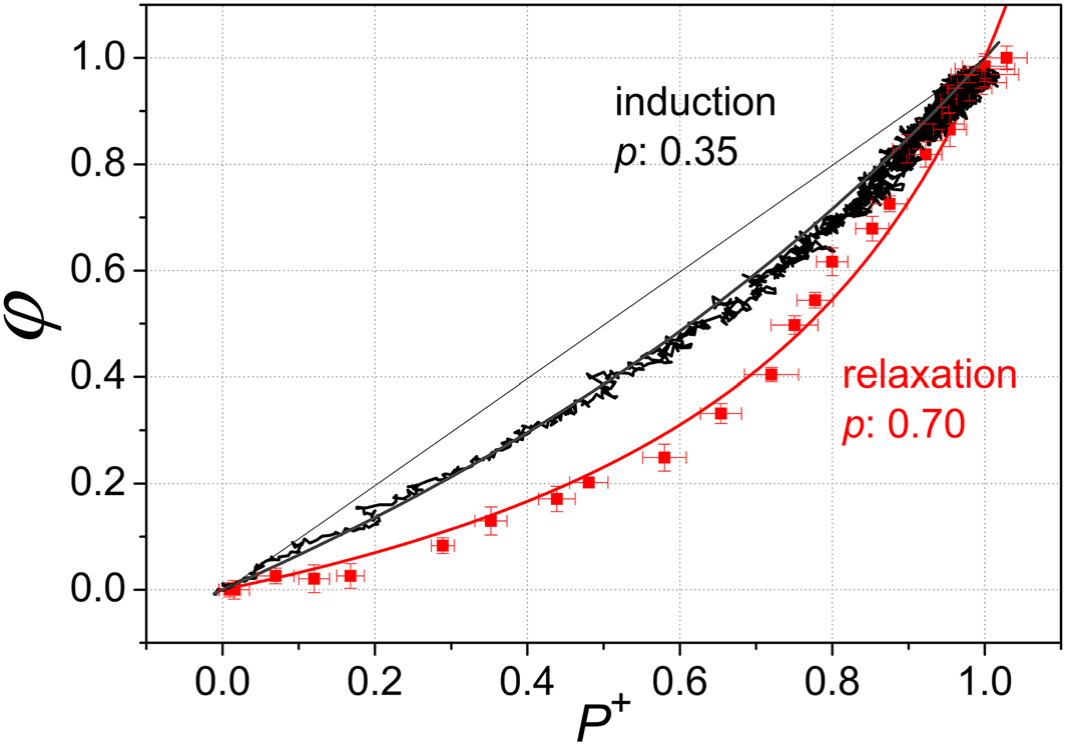
Demonstration of large hysteresis due to the increased difference between the kinetics of fluorescence yield and closure of the PSU during induction and relaxation. The bacteria were harvested in the early phase of their growth (24 h after inoculation). Otherwise the experimental conditions and evaluation of the data were the same as in Fig. 4.

The curvature of the data measured in the light is smaller than that measured in the dark which would correspond formally to different *p* values: 0.3 during induction and 0.5 during relaxation. The magnitude of the hysteresis probably depends on the physiological condition of the bacteria, as younger (24 h) cells demonstrate a more pronounced effect: $p = 0.35$ for induction and $p = 0.70$ for relaxation (Fig. 5). The fit to the Joliot model is not perfect as it shows systematic deviation from the measured points. It reflects that the hyperbola is not symmetric i.e. it does not cut the diagonal of slope 1 at equal angles [see Eqs. (4) and (6)].

### Mathematical results beyond Joliot approach

#### Generalised mean-field models

In the exciton wandering model with relations in Eqs. (2) and (5) we use more elaborated approaches for the multisite correlations, $G_k$, in Eq. (2) (we also set $k_I=1$). The differences with the Joliot model (standard mean-field approach) are the following. (1) The exciton has a limited hopping length, so that it can visit only the RCs in nearest neighbour position. (2) The life-time of the exciton is limited, it can make $n < \infty$ steps. Note that *n* and the hopping probability *p* are linked since the average number of steps of the exciton (corresponding to its life-time) is given by a combination (see Eq. (27) in “Mathematical methods”), which has its maximal value: $\langle n \rangle_{\max } =(1-p^n)/(1-p)$. Furthermore, in the actual calculation for simplicity we assume that the RCs occupy a regular lattice, having a coordination number, *z*. (In the numerical work it is a square lattice with $z=4$.)

Using this settings the *k*-site correlations are defined by the average over all possible *k*-step walks:

7 $$\begin{aligned} G_k=\langle \sigma_{i_1}\sigma_{i_2} \ldots \sigma_{i_k}\rangle =\frac{1}{Nz^{k-1}} \sum_{i_1 \curvearrowright i_2 \curvearrowright \ldots \curvearrowright i_k}\sigma_{i_1}\sigma_{i_2} \ldots \sigma_{i_k}. \end{aligned}$$

Note, that in the sum in Eq. (7) the same site can be visited several times. In this case using the identity: $(\sigma_i)^s=\sigma_i,\quad s=2,3,\ldots$, one obtains a set of *j*-site reduced correlations, $x_j$ with $2\le j < k$, such that

8 $$\begin{aligned} G_k =\sum_{j=2}^k c_j^{(k)}x_j, \end{aligned}$$

where $c_j^{(k)}$ is the fraction of *k*-step random walks which have visited *j* different sites. In the following we use the notation: $x=x_1=P^+$ for the one-site function.

The multi-site correlations are expected to be different during induction and relaxation. During relaxation the spontaneous opening of the RCs is uncorrelated in time, so that multi-site correlations depend solely on the density, *x*. For example the two-point correlation function is given by: $\langle \sigma_{i_1}\sigma_{i_2}\rangle_{\mathrm{rel}}=\langle \sigma_{i_1}\rangle \langle \sigma_{i_2}\rangle =x^2$ and similarly $x_j=x^j$. This type of description is called the lattice mean-field (LMF) method. On the contrary, during induction the exciton makes jumps to nearest-neighbour sites, which creates short-range correlations. For example the two-point function during induction generally satisfies: $\langle \sigma_{i_1}\sigma_{i_2}\rangle_{\mathrm{ind}} \ge \langle \sigma_{i_1}\rangle \langle \sigma_{i_2}\rangle =x^2$. As a consequence $\varphi_{\mathrm{ind}} \ge \varphi_{\mathrm{rel}}$, in agreement with the experimentally observed hysteresis in the fluorescence yield. In induction in order to describe this type of bunching effect of closed RCs we use the cluster mean-field (CMF) method. In the CMF approach the $x_j$-s are expressed in terms of one-site, $\langle \sigma_{i_1} \rangle =x$, and two-site correlations, $\langle \sigma_{i_1}\sigma_{i_2}\rangle =x_2$, where $i_1$ and $i_2$ are nearest neighbours.

**Figure 6:**
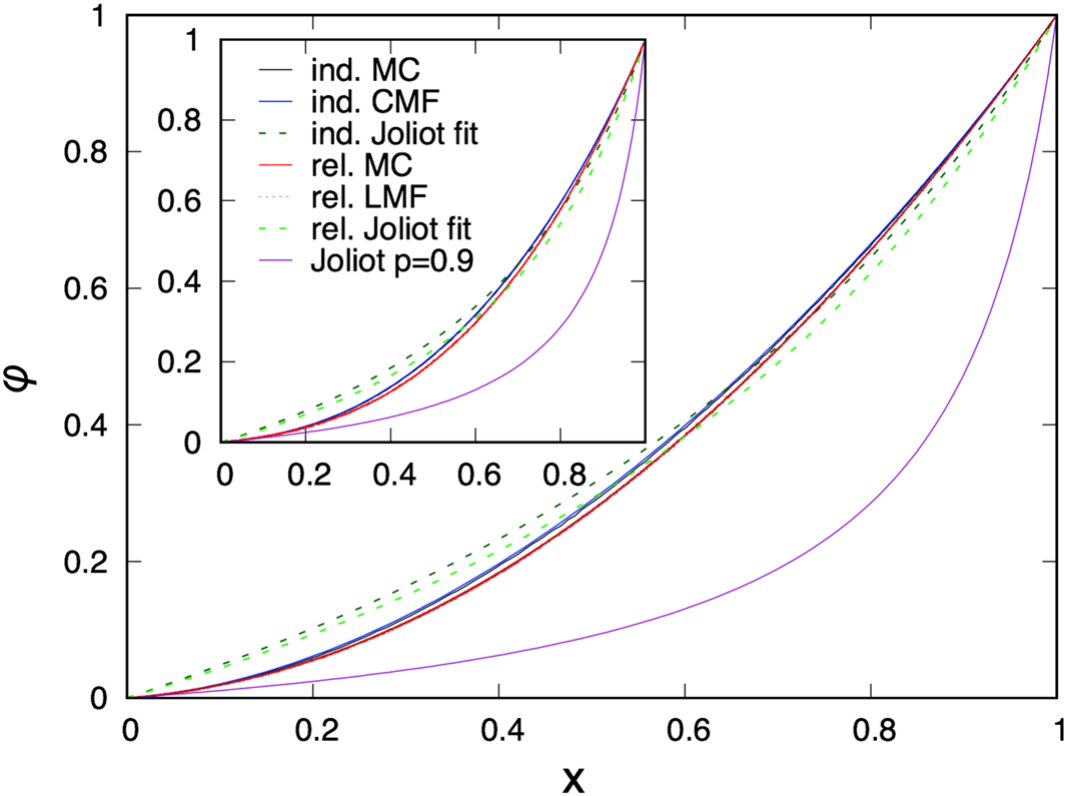
Fluorescence yield as a function of the fraction of closed RCs calculated during relaxation (LMF calculation and MC simulations) and during induction (CMF calculation and MC simulations) at a hopping probability $p=0.9$ for $n=2$ (main panel) and $n=3$ (inset). In both cases the best fit (with *p*) of the Joliot theory, as well as the result with a fixed $p=0.9$ is also presented.

#### Lattice mean-field approach

In the LMF approximation we have $x_j=x^j$ and the fluorescence yield is given as an *n*-th degree polynomial of *x*, which is illustrated in the insets of Fig. 6 for $n=2$ and 3 at a hopping probability $p=0.9$. At $n=1$ the relation is linear, $\varphi =x$, which is modified for $n=2,3,\ldots$, when exciton wandering is taken into account.

**Figure 7:**
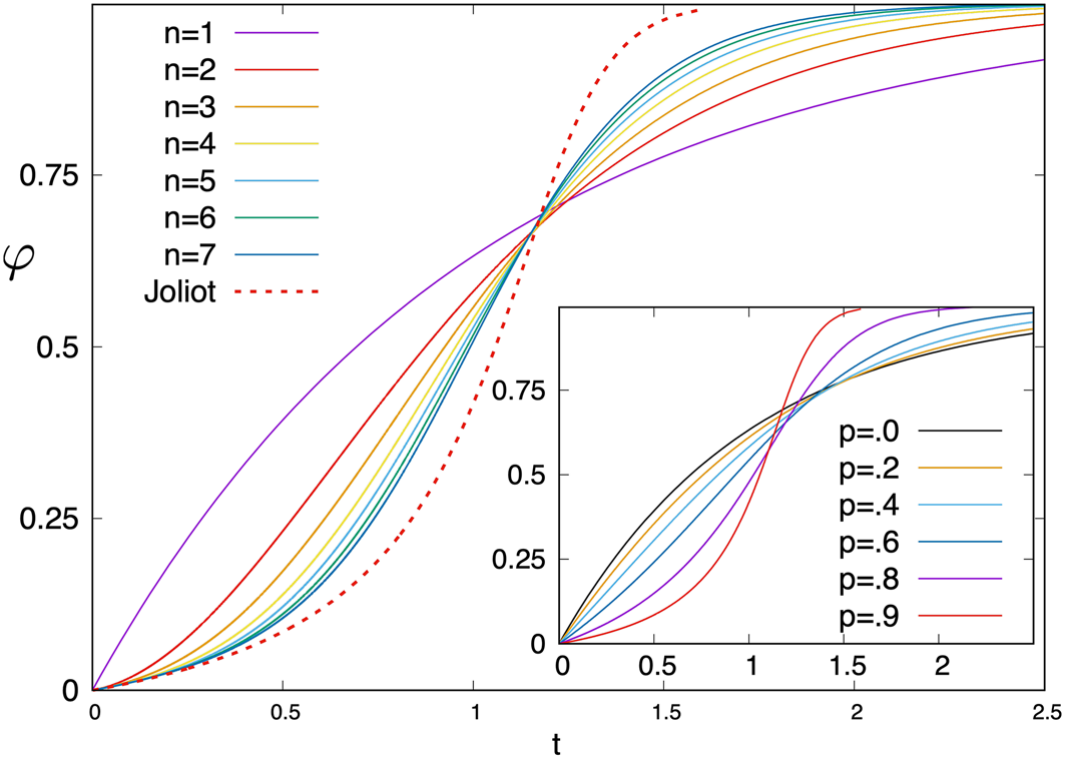
Time-dependence of the fluorescence yield calculated by the LMF approximation for various values of *n* at a hopping probability $p=0.9$. The result of the Joliot theory with the same *p* is shown for comparison. Inset: Time-dependence of the fluorescence yield in the Joliot model for different values of the hopping probability.

The time-dependence of the fluorescence yield is obtained by performing the integral in Eq. (13), either analytically for $n=1,2,3$, see in Eqs. (14–16), or numerically for $n>3$. The kinetics, $\varphi (t)$ for different values of *n* at the hopping probability $p=0.9$ is shown in Fig. 7. It is seen in this figure, that $\varphi (t)$ for $n \ge 2$ in the starting time-period is convex, which turns to concave at an inflection point. The curves with different values of *n* cross each others approximately at the same (inflection) point. The sigmoidicity of the fluorescence induction kinetics is overestimated by the Joliot theory with the same $p=0.9$.

#### Cluster mean-field approach

**Figure 8:**
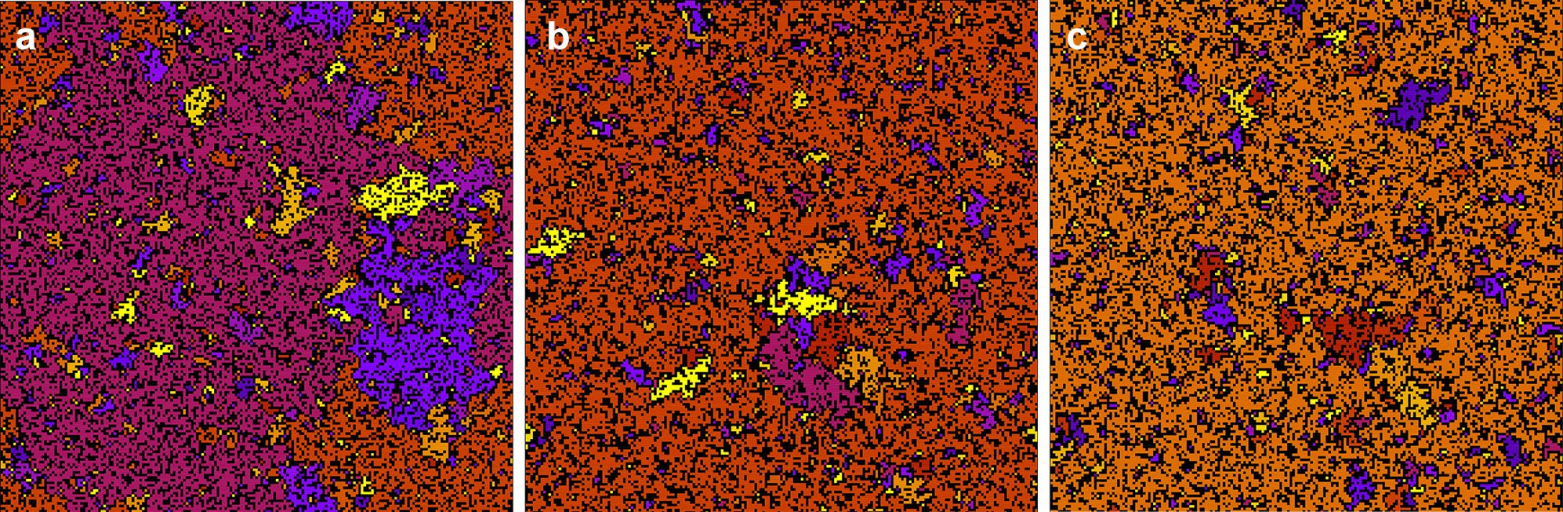
Typical cluster structures of RCs on a $200 \times 200$ square lattice at an occupation probability, $x=0.594(1)$, slightly above the site-percolation threshold. Left panel: uncorrelated percolation, corresponding to the structure during relaxation with $n=1$. Middle panel: during induction with $p=0.9$ and $n=2$. Right panel: during induction with $p=0.9$ and $n=3$. Sites with the same colour represent connected clusters of closed RCs.

During induction exciton wandering creates a bunching effect of closed RCs which is illustrated in Fig. 8. Here typical cluster structures of the RCs are presented on the square lattice at an occupation probability, $x=0.594(1)$, slightly above the site-percolation threshold ($x_{\textit{perc}}=0.5927460$^24,25^) with $n=1,2,$ and 3. For $n=1$, which corresponds to uncorrelated percolation and represents the state of the system during relaxation, the formation of a giant *fractal* cluster is visible. For $n=2$ and $n=3$, which illustrate the state of the system during induction the giant cluster is visibly *compact*. This is explained by the effect of exciton bunching, which causes a decrease in the critical percolation threshold so that the system is in the super-critical phase for the given value of *x*.

**Figure 9:**
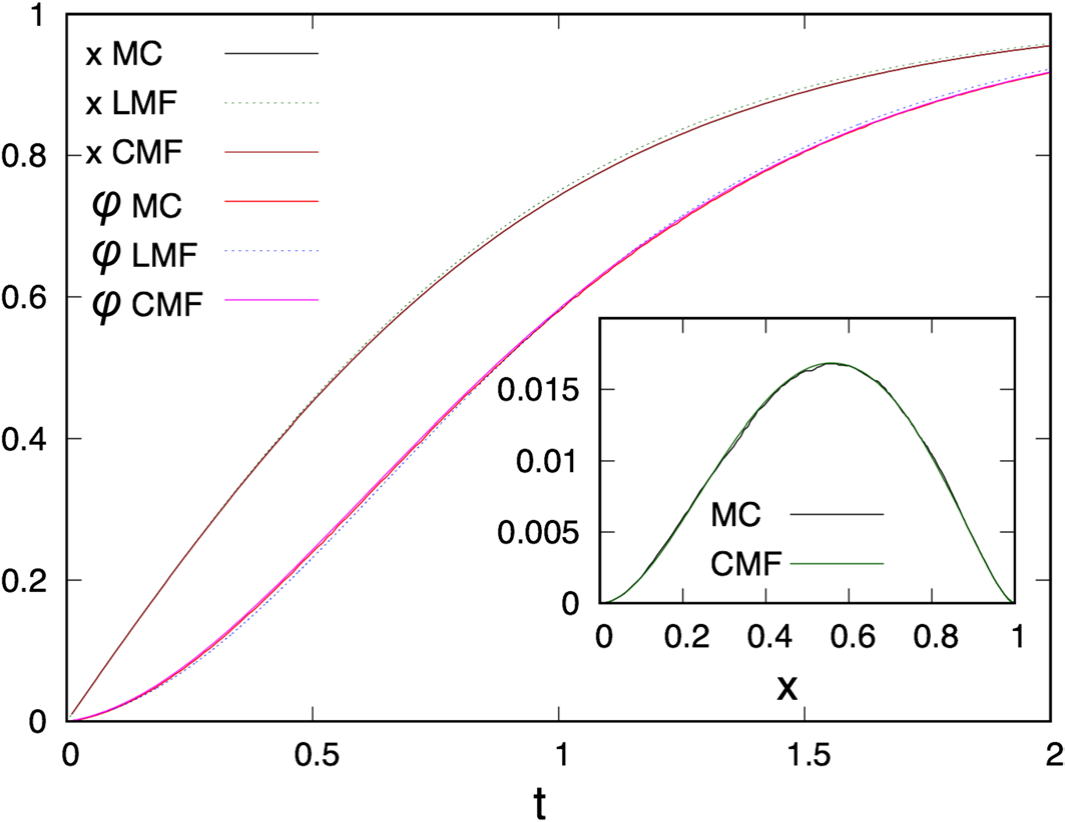
Dynamics of the order-parameter, *x*(*t*), and the fluorescence yield, $\varphi (t)$, calculated for a hopping probability $p=0.9$ and for $n=2$. Results of the LMF and CMF approaches are compared with MC simulations during induction. Inset: Connected nearest-neighbour correlation function $\tilde{x}_2=x_2-x^2$ as a function of *x* for $p=0.9$ and for $n=2$. The CMF calculations perfectly overlap with the results of MC simulations.

We have calculated the nearest-neighbour correlation function, $x_2=\langle \sigma_{i_1}\sigma_{i_2}\rangle$ through MC simulations and compared the results with its uncorrelated value: $\langle \sigma_{i_1}\rangle \langle \sigma_{i_2}\rangle =x^2$. The difference, the connected correlation function $\tilde{x}_2=x_2-x^2$ is shown in the inset of Fig. 9 as a function of *x*, which is certainly not negligible, for $x \le 0.6$ their relative weight is about $10\%$. We have checked that a similar trend is present for larger values of $n=3$ and 4, and that the correlations for nearest neighbors are larger than those between more remote sites (i.e. those having a distance of two or three lattice units).

Based on this observation we introduce the CMF approach, in which the bunching of closed RCs is taken into account through one more parameter, the nearest-neighbour correlation function, $x_2$. $x_2$ is obtained through the solution of the dynamics of a two-site cluster and the correlations which involve more sites are expressed in terms of two-site and one-site functions. For example the three-site function in this approach is given by:

9 $$\begin{aligned} \langle \sigma_{i_1}\sigma_{i_2}\sigma_{i_3}\rangle \approx \frac{\langle \sigma_{i_1}\sigma_{i_2}\rangle \langle \sigma_{i_2}\sigma_{i_3}\rangle }{\langle \sigma_{i_2}\rangle }=\frac{(x_2)^2}{x}, \end{aligned}$$

while the general result is given in Eq. (17). Having the analytical results in Eqs. (22) and (24) we have calculated $\tilde{x}_2=x_2-x^2$, which is plotted for $p=0.9$ as a function of *x* in the inset of Fig. 9. Comparing it with the numerical values, obtained by MC simulations during induction an almost perfect agreement is obtained.

We have also calculated the time-dependence of the order-parameter, *x*(*t*), and that of the fluorescence yield $\varphi (t)$, in the CMF approach, the results are shown in Fig. 9 together with those calculated by the LMF approach as well as with MC simulations during induction. It is seen in this figure that $x(t)>\varphi (t)$, which is due to exciton wandering. The results of CMF perfectly fit the MC simulations, at least within the numerical accuracy of the latter method. On the contrary the results of the LMF methods show small, but non-negligible differences. The LMF results overestimate *x*(*t*) in particular for large *t*. For the fluorescence yield the LMF approach underestimates it at small *t*, but overestimates it for large *t*.

Finally, we calculate the relation between the fluorescence yield $\varphi$ and the fraction of closed RCs, *x*, and the results for the square lattice are presented in Fig. 6 for $n=2$ (main panel) and $n=3$ (inset), at a hopping probability $p=0.9$. Here we have made the calculations both during relaxation, when the LMF results are compared with MC simulations and with the (best fit of) the Joliot theory, and during induction, when the CMF results are compared with MC simulations and with the (best fit of) the Joliot theory. For $n=3$ the fluorescence yield from Eq. (2) is given by:

10 $$\begin{aligned} \varphi =(1-p)x+(1-p)px_2+p^2 \left( \tfrac{1}{4}x_2+\tfrac{3}{4}x_3\right) , \end{aligned}$$

where the three-site function is written as $x_3 \approx (x_2)^2/x$, in agreement with Eq. (9).

As seen in Fig. 11 the analytical calculations agree very accurately with the MC simulations, both during relaxation (in which case the uncorrelated structure of the RCs perfectly fit with the similar assumptions of the LMF approach) and during induction (in which case the bunching effect of the closed RCs are well modelled in the CMF approach). In the curves there is a hysteresis, at the same value of *x* the fluorescence yield is larger during induction, than during relaxation, which is due to the bunching effect. The hysteresis increases with larger value of *n*, since the bunching effect is also larger in this case. Concerning the Joliot theory the curve with $p=0.9$ fits only the starting part of the curves for small *x*, but deviates considerably for larger *x* values. We can have an overall better description, if we set *p* as a free parameter. Then, by using different best fit parameters during induction and relaxation the agreement with the measured curves becomes better, although still far less satisfactory, than the LMF and the CMF results.

## Discussion

The transfer of the excitons to the RC is extremely efficient as almost every photon of the absorbed light in the antenna is used by the RC^26^. The extreme efficiency of light utilization supports the assumptions used above: the exciton is trapped or reflected by collisions with open or closed RCs, respectively and the redirected exciton can visit several other RCs. Indeed, the RC of the *cycA* mutant acts accordingly: the open state PQ_A_ is a perfect trap and the closed state P^+^Q_A_^–^ is a perfect reflector of the incoming excitons. In this variant, the oxidized dimer P^+^ of the closed RC does not allow any additional charge separations including the short lived P^+^BPheo^–^ radical pair. There is no known exciton-radical pair equilibrium, whose “reverse reaction” would be required to apply standard (homogeneous kinetic) models^11^. This introduces the phenomenological concept of imperfect traps for open ($0.25 \pm 0.05$) and closed ($0.40 \pm 0.05$) RCs (*R. rubrum*) to describe the characteristics (e.g. the initial and maximum levels) of the fluorescence induction^27^. The probability of redirection of the exciton from the open RC to the antenna has been estimated between 5–30% in various purple bacteria^11^. In contrast, the exciton walking approach does not need this ad hoc assumption, instead, it considers the RC as a perfect trap (for photochemistry) or reflector (for migration) of the excitons.

A long-standing question is how the efficiency of the bacterial antenna can be so high at ambient temperature given that it is a partly disordered biological system. The structural data from atomic force^28,29^ and cryo-electron microscopy^30^ and functional results from two-dimensional electron spectroscopy and related calculations^31^ clearly demonstrate the close packing of the BChl complexes and the strong coupling, respectively, which are the necessities for exciton formation. One can ask whether the funnelling of the excitation energy to the RC occurs through random hops or straight walks of the exciton? The interaction among the chromophores within the PSU can be so high that even the signs of quantum coherence may appear^32,33^. Currently, the temptation is large to attribute the quantum coherence observed in the antenna system of photosynthetic organisms to be similar to that in quantum computers^34^. However, the energetic coupling among the PSUs is not so large as among the chromophors within the PSU. The smaller connectivity permits a random walk rather than a direct walk of the excitons to the nearest open RC. This is why we pictured the movement of the excition as a random (incoherent) hopping process. The excitation at an arbitrary site of the antenna does not find an optimal route to the nearest open RC but has to waste time through random hopping. The point is addressed that the random hopping of excitation fits better to the structure of and interaction within the bacterial antenna system than the brute-force approach of full quantum models.

The random walk approach applied in this study drops two essential simplifications which limit the validity of the Joliot theory. (1) The exciton redirected from a closed RC can visit any RC (independent of their relative locations) with probability *p* (Joliot parameter) or with connectivity parameter $J = p/(1-p)$. This is a disputed assumption of the Joliot model as the rate of energy transfer between donor and acceptor chromophores has strong distance-dependence (see the inverse power 6 dependence of the rate constant via dipole–dipole interaction in the Förster mechanism). The transfer (hop) to the neighboring RC is more probable than to a distant RC. The real motion of the exciton is adequately treated by a random walk on the network of the RCs as used in our model. (2) The distribution of the closed RC is taken randomly at any moment of the kinetics. However, this assumption is true during the relaxation only and fails during the induction. On the one hand, the fraction of closed RC in the relaxation process is controlled by the chemical re-reduction of P^+^ and the distribution remains always random during the decay. On the other hand, when the RCs are closing progressively under a continuous excitation (induction), the distribution of the closed RCs will not be random due to bunching effects: an open RC has higher chances to become closed when its neighbors are already closed. The distribution will differ from the Poisson distribution, and will depend on the degree of saturation (i.e. on the time). The simultaneous fluorescence and absorption change kinetics observed both during induction and during relaxation indicate clearly the limits of the standard theory and the experimental manifestation of the bunching effect (Figs. 2 and 3). The concavities of the $\varphi (x)$ curves were different: it was smaller during induction than during relaxation and the difference (hysteresis) seemed to be dependent on the physiological state (age) of the bacteria.

Here we used an exciton migration model in which the possible pathways of the exciton were represented with different approximations. In the homogeneous kinetic model the exciton could hop to any RC irrespective of its distance and position and the PSU dynamics was treated in the (one-site) mean-field level. In the LMF approach the exciton hoping was restricted to nearest neighbour RCs and the same closed RC could be visited several times, but the PSU dynamics was still in the mean-field level. Finally, in the CMF approach, while the exciton wandering respects the local topology of the RCs, the PSU dynamics were treated at the (two-site) cluster level. In this way bunching of closed RCs during induction was taken into account and the experimentally observed hysteresis could be successfully explained. The basic ingredients and approximations which were used in the different approaches are summarised in Table 1.

**Table 1:** Basic ingredients involved in the different approaches.

	Joliot-theory	LMF	CMF
Density of closed RCs	Yes	Yes	Yes
Dynamics of *x*	Yes	Yes	Yes
Lattice topology	No	Yes	Yes
Multiple visits of sites	No	Yes	Yes
Bunching of closed RCs	No	No	Yes
Hysteresis	No	No	Yes

**Figure 10:**
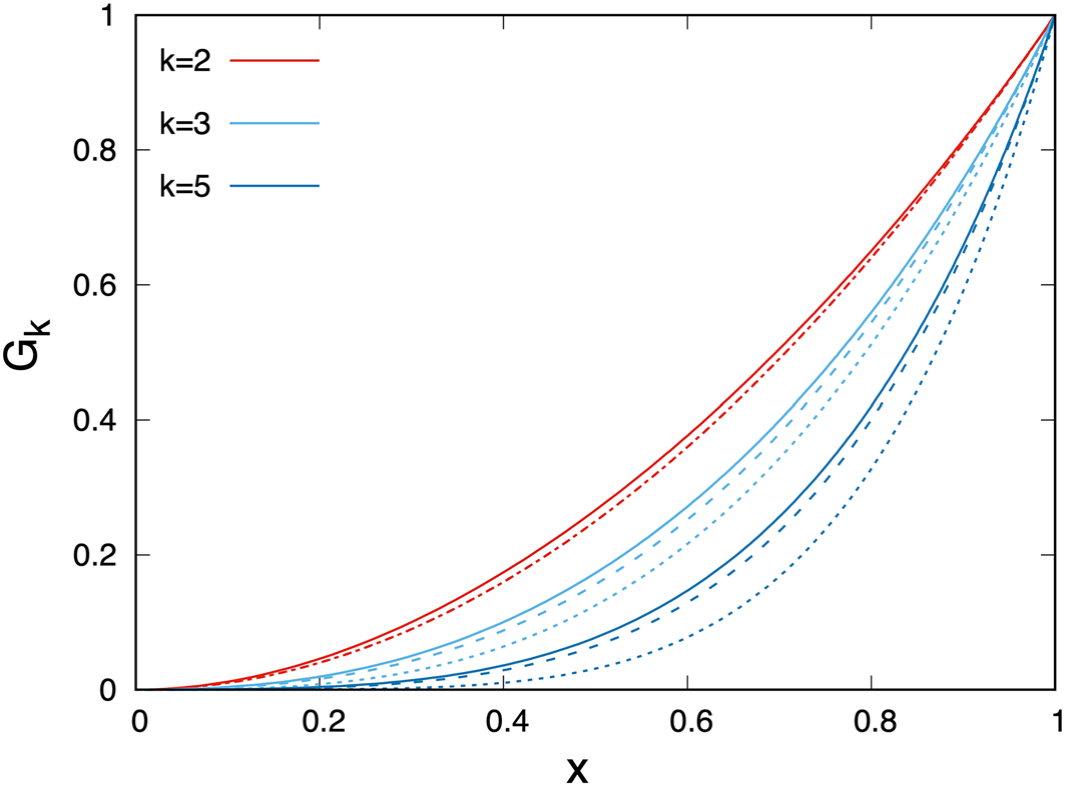
*k*-site correlations versus fraction of closed RCs calculated in the different approaches at a hopping probability $p=0.9$. Dotted line: Joliot-theory, dashed line: LMF approach, full line: CMF approach.

The multi-site correlation functions, $G_k=\langle \sigma_{i_1}\sigma_{i_2} \dots \sigma_{i_k}\rangle$, were introduced as fundamental quantities of the theoretical treatment [see Eq. (7)]. $G_k$ is the fraction of such *k*-step random walks (modelling exciton wandering), which visit (nearest neighbour) closed RCs. The approximate representation of $G_k$ in the different approaches is described in details in Sect. 4.2. The *x* dependence of $G_k$ is illustrated in Fig. 10 for different values of *k* at a hopping probability $p=0.9$. As a general rule $G_k$ is larger if the exciton finds more closed RCs at the nearby steps. Therefore $G_k$ is a monotonously increasing function of *x*. For a given value of *k*, $G_k$ is the smallest in the Joliot theory, in which multiple visits of the same closed RC don’t take place. Comparing the results from the CMF and LMF approaches, the former is somewhat larger due to the bunching effect. Based on the multi-site correlations, some essential quantities can be calculated, such as the absorption cross-section of the RC (due to the presence of closed RCs in the neighborhood) and the average number of exciton steps during migration.

**Figure 11:**
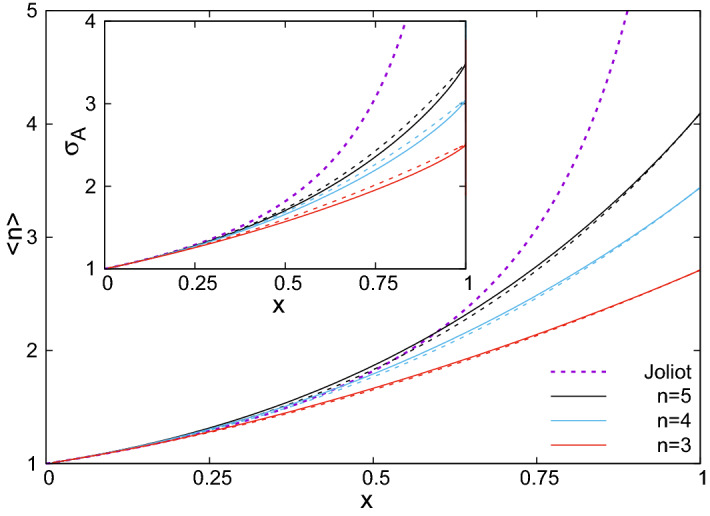
Average number of exciton steps as a function of the fraction of closed RCs at a hopping probability $p=0.9$ calculated with the CMF approach (full line) and with the LMF approach (dashed line), for different maximal number of steps, *n*. With dotted line result of the Joliot-theory ($n \rightarrow \infty$) is presented. Inset: absorption cross section as a function of the fraction of closed RCs.

The absorption cross section, $\sigma_A$, calculated in the different approaches is shown in the inset of Fig. 11 at a hopping probability $p=0.9$. It is a monotonously increasing function of *x*, as more and more RCs will be closed in the vicinity. It is also increasing with *n*, when more exciton steps can be made. Since in the Joliot theory *n* is unlimited, the corresponding absorption cross section is much larger, than those for finite values of *n*. Having the same value of *n*, $\sigma_A$ is somewhat larger during relaxation (which corresponds to the LMF approach), than during induction (described with the CMF method). Due to bunching the exciton stays longer on closed RCs in the latter process, and has smaller probability to reach an open one.

The average number of exciton steps, $\langle n \rangle$, calculated in the different approaches is shown in the main figure of Fig. 11 at a hopping probability $p=0.9$. It is seen, that $\langle n \rangle$ is a monotonously increasing function of *x* and has its maximum at $x=1$: $\langle n \rangle_{{\max}}=(1-p^n)/(1-p)$. The general shape of the curves is similar to that of the absorption cross section in the inset of Fig. 11, with the difference, that for a given *n*, its average value is larger during induction (CMF method), than during relaxation (LMF approach). Indeed, due to bunching, the exciton finds closed RCs with higher probability in the former process.

Any changes of the physiological state reflect adaptation of the bacterium to the variable environmental conditions with the goal of establishing a fine balance against several requirements. The changes of the light intensity result in changes of the antenna organization and exciton migration^6,23^. Under light-limiting conditions, the light must be collected with higher efficiency by increase of the LH2 antenna size and of the connectivity of the PSUs. At higher light intensity, photobleaching becomes the bottleneck. The fraction of closed RCs comes closer to saturation resulting in the increase of the absorption cross section of the RCs (inset of Fig. 11) and in the number of steps (and thus the lifetime) of the migrating excitons (Fig. 11). These effects enhance the probability of BChl triplet formation and make the bacterium more vulnerable to photooxidation. The response of the cell to these conditions is the reduction of the energetic coupling of the PSUs by loosing the antenna structure including setting spacers between the LH complexes. The loose-fitting core complex may facilitate the diffusion of the quinone that shuttles electrons and protons between RC and cyt *bc*_1_ complex^35,36^. Tentatively, we assigned the observed dependence of hysteresis on the duration of the cultivation of the bacteria to changes of the membrane packing. Further work is required to confirm and understand the effect.

The intactness of the cells had special importance in this study as all earlier works referred to chromatophores. While the optical signal was disturbed by light scattering due to the larger sizes of the cells, we were able to diminish its effect and obtain optical signals with quality close to those obtained from chromatophores. By use of whole cells instead of chromatophors prepared by invasive biophysical and biochemical methods, we could preserve the physiological state of the bacterium.

The observed hysteresis (bunching effect) may include unexpected and interesting manifestation of a memory function. That is, the induction process is influenced by a sort of memory of the way the given state was prepared. Near-neighbour correlations are induced between the closed RCs, thus their distribution is not fully random. This correlation can be taken as the expression of the memory of the state. On the contrary, the relaxation process is controlled by spontaneous re-reduction of P^+^ (via charge recombination or external electron donor), thus the distribution of the closed RC-s is completely random and uncorrelated. No “memory function” can be introduced.

For a fixed fraction of closed PSUs (*x*), it is interesting to see how the fluorescence yield depends on the connectivity of the units (Figs. 4 and 5). As expected, the energetic coupling (*p*) decreases the fluorescence yield but the decline depends on the pattern of the distribution of the closed RCs (hysteresis): it is smaller in induction than during relaxation under otherwise identical conditions. The correlated clusters of closed centers during the induction phase results in larger fluorescence than the uncorrelated clusters during the relaxation phase. In the case of too much excitation (including not only high exciton density but a large fraction of closed RCs, as well), the fluorescence can be considered as a valve function of energy dissipation that is useful for photoprotection^37^. The observed phenomenon of hysteresis may reveal new aspects of the competition between harvest and dissipation of light energy and can serve as a fine tuning mechanism of the light utilization in the antenna.

## Methods

### Materials and experimental methods

#### Cytochrome c less bacterial mutant strain and chemicals

The *cycA* mutant was constructed from a wild type strain of purple nonsulfur photosynthetic bacterium *Rhodobacter (Rba.) sphaeroides* as described earlier^38^. Strain JS2293$\Delta$, containing an in-frame deletion of the *cycA* gene encoding cytochrome *c*_2_ in *Rba. sphaeroides*, was genetically constructed essentially as described previously^39^. *Escherichia coli* strains were grown at 37 °C in LB medium^40^ supplemented with antibiotics when appropriate; kanamycin (50 $\upmu$g mL^–1^) and ampicillin (100 $\upmu$g mL^–1^). *Rba. sphaeroides* strains were grown aerobically at 30 °C in YCC medium^41^ supplemented when appropriate with kanamycin (50 $\upmu$g mL^–1^). Conjugal transfer of strains from *E. coli* to *Rba. sphaeroides* was performed as described previously, and counter-selection against S17-1 donors was achieved by addition of tellurite (100 $\upmu$g mL^–1^)^42^. The cyt *c*_2_ mutant bacteria were cultivated in a half filled Erlenmeyer flask plugged by rolls of cotton wool (semiaerobic conditions) on a shaking plate in the dark. The increase of concentration of bacteria saturated three days after the inoculation and samples could be taken in different phases of the bacterial growth (see^43^).

To inhibit the interquinone electron transfer after flash excitation, terbutryne was used in 120 $\upmu$M concentration. The herbicide terbutryn has proved to be highly efficient even in whole cells of bacteria to block the Q_A_^–^Q_B_$\rightarrow$ Q_A_Q_B_^–^ electron transfer in the acceptor quinone complex of the RC by competition with the pool quinones for the same secondary (Q_B_) binding site.

#### Optical measurements

The transient changes of absorption and fluorescence of the intact cells were generated by high power (2 W) laser diodes using variable flash durations. Flashes that were approximately 1 ms in duration were energetically sufficient to cause the gradual closure of all of the mutant RCs. Red (wavelength 804 nm) or blue (wavelength 450 nm) laser diodes were applied to excite the BChl dimer P of the RC directly via BChls of the LH2 or indirectly through accessory pigments (e.g. carotenoids). The different excitation modes delivered very similar results.

*Absorption*: Because the light-induced oxidation of the RC dimer induces an electrochromic shift in the absorption band of the nearby monomeric BChl^44^, the kinetic status of the oxidized dimer (P^+^) was tracked by measurement of the absorption change at 790 nm. The weak measuring beam was chopped for long periods of time by a mechanical shutter to avoid the excitation of the sample during both induction and relaxation. As the magnitude of the absorption change proved to be small ($\Delta$*A* $\sim$ 1 mOD), the absorption kinetics were acquired as averages of several (up to 64) scans to reduce the statistical error. The rate of repetition of the flashes had to be fitted to the complete relaxation of the P^+^Q_A_^–^ charge separated state ($\sim$ 20 s). More (128) scans were needed to measure the absorption changes during relaxation where the small signal-to-noise was overlapped by the slow drift of the baseline in the prolonged time scale of about 10 seconds.

*Fluorescence*: The home-built experimental set-up (BChl fluorometer) and the data processing of fluorescence of intact cells have been described in detail^45^. The fluorescence (wavelength centered at 900 nm) from sample in $3 \times 3$ mm quartz cuvette was measured during the excitation during induction mode and detected by testing flashes in relaxation mode. The fluorescence quanta emitted in the direction perpendicular to the actinic light beam were detected by a near-infrared-sensitive, large-area (diameter 10 mm), and high-gain Si-avalanche photodiode (APD; model 394-70-72-581; Advanced Photonix Inc., USA, working resistance 1.5 k$\Omega$). A long pass filter (RG 850, Schott) was used to protect the detector from scattered light of the laser and to cut off fluorescence emission from the other pigments than BChl and the base plate. Solutions of extracted BChl or IR-806 dye (Sigma) served as references for fluorescence yield measurements and to correct for any deviations from the step function (rectangular shape) and for large-scale fluctuation of the laser diode excitation. The reference signal was adjusted to the same intensity as that of the fluorescence to avoid the possible artefact coming from the nonlinearity of the response of the detector at high light intensity.

The extreme values of the induction kinetics of fluorescence (*F*) were determined experimentally as follows. The constant part of the fluorescence rise (*F*_0_) was obtained by interception of the initial data (approximated by straight line) and the vertical axis at $t = 0$ and the maximum fluorescence (*F*_max_) by the saturating value of the induction. The normalized variable fluorescence was derived as $\varphi$ = (*F*-*F*_0_)/(*F*_max_-*F*_0_). The fluorescence of the sample during relaxation was probed by a couple ($\sim$ 15) of intense but short (5 $\upmu$s) laser flashes. The non-exciting character of the testing flashes was checked before each experiment.

All measurements were performed at room temperature (20–25 °C).

### Mathematical methods

The multi-site correlation function $G_k$, is the fraction of such *k*-step random walks which visit (nearest neighbour) closed RCs, see in Eq. (7). In the *Joliot theory* multiple visits of the same site is excluded and the multi-site correlations are approximated as products of one-site functions as $G_k=x^k$.

#### Lattice mean-field approach

In the LMF approach the exciton hops on nearest-neighbour lattice sites, multiple visits of the same site is allowed and the local topology of the lattice is encoded in the weights, $c_j^{(k)}$, $j=2,3,\ldots ,k$. Reduced multi-site correlations are approximated as product of one-site functions:

11 $$\begin{aligned} G_k = c_2^{(k)}x^2 + c_3^{(k)}x^3 + \cdots +c_k^{(k)}x^k=\sum_{j=2}^k c_j^{(k)}x^j. \end{aligned}$$

**Table 2:** List of $C_j^{(k)}/4$, see text. These parameters appear as the weights of the polynomials in Eqs. (8) and (12).

k	2	3	4	5	6	7	8	9	10
$\hbox {j}=2$	1	1	1	1	1	1	1	1	1
3		3	6	12	18	30	42	66	90
4			9	26	72	161	338	690	1317
5				25	94	319	890	2335	5668
6					71	318	1256	4066	12,325
7						195	1026	4515	16,434
8							543	3232	15,692
9								1479	9942
10									4067

The weights are calculated through random walk statistics using the parameterisation:

12 $$\begin{aligned} c_j^{(k)}=C_j^{(k)}/z^{k-2}, \end{aligned}$$

where $C_j^{(k)}/z$ is the number of $(k \ge 2)$-step random walks, which have visited $2 \le j \le k$ different sites, where the walker arrives to the lattice at the first step. For the square lattice with $z=4$ the first few terms of $C_j^{(k)}$ are given in Table 2.

The dynamics of *x* is calculated from Eq. (5) and with separation of the variables it is given by ($k_I=1$):

13 $$\begin{aligned} \int_0^x \frac{\text {d} x'}{1-\varphi (x')} = t. \end{aligned}$$

Here the denominator is a polynomial of $x'$, and thus can, in principle, be integrated for all values of *n*. For the first three values of *n* these are given by:

14 $$x(t)=1-\exp (-t),\quad n=1,$$

15 $$x(t)=\frac{\exp [(1+p)t]-1}{\exp [(1+p)t]+p},\quad n=2,$$

16 $$\begin{aligned}t=& \frac{(1+1.5p)/\sqrt{2}}{(1+p+3/4p^2)}\left[ \arctan \left( \frac{1.5px +1}{\sqrt{2}}\right) -\arctan \frac{1}{\sqrt{2}}\right] \nonumber \\&-\frac{1}{2(1+p+3/4p^2)}\ln \frac{|1-x|^2}{|1+px +3/4(px)^2 |},\quad n=3. \end{aligned}$$

#### Cluster mean-field approach

In the CMF approach the exciton hops on nearest-neighbour lattice sites, multiple visits of the same site is allowed and the local topology of the lattice is encoded in the weights, $c_j^{(k)}$, $j=2,3,\dots ,k$. Multi-site correlations are expressed in terms of two-site ($x_2$) and one-site functions:

17 $$\begin{aligned} G_k= & {} c_2^{(k)}x_2 + c_3^{(k)}\frac{(x_2)^2}{x} + \cdots +c_k^{(k)}\frac{(x_2)^{k-1}}{x^{k-2}} \end{aligned}$$

The basic correlations in the CMF approach are calculated for a two-site cluster, in which case the occupation probabilities of the different configurations are given by: $P_{\circ ,\circ }$, $P_{\circ ,\bullet }$, $P_{\bullet ,\circ }$ and $P_{\bullet ,\bullet }$. Here $P_{\circ ,\bullet }=P_{\bullet ,\circ }$ due to symmetry and $P_{\circ ,\circ }+2P_{\circ ,\bullet }+P_{\bullet ,\bullet }=1$, due to normalisation. Thus we have two independent parameters: $x=P_{\circ ,\bullet }+P_{\bullet ,\bullet }$ and $x_2=P_{\bullet ,\bullet }$, so that $P_{\circ ,\bullet }=x-x_2$ and $P_{\circ ,\circ }=1-2x+x_2$.

The time-dependence of the one-site function is given by:

18 $$\begin{aligned} -\frac{\text {d} P_{\circ }}{\text {d} t}=P_{\circ }+p'zP_{\circ ,\bullet }, \end{aligned}$$

where $p'=p/z$ is the hopping probability from the  site of the  cluster, having *z* different orientations in the lattice. The time-derivative of the two-point function, $P_{\circ ,\circ }$, involves the three-point function, $P_{\circ ,\circ ,\bullet }$, in which the  site can be in $2(z-1)$ relative positions with respect to the  cluster:

19 $$\begin{aligned} -\frac{\text {d} P_{\circ ,\circ }}{\text {d} t}= & {} 2P_{\circ ,\circ }+2p'(z-1)P_{\circ ,\circ ,\bullet } \end{aligned}$$

and use the approximation $P_{\circ ,\circ ,\bullet }\approx P_{\circ ,\circ }P_{\circ ,\bullet }/P_{\circ }$.

Introducing the notations: $P_{\circ }=q$, $P_{\circ ,\circ }=u$, so that $P_{\circ ,\bullet }=q-u$, the equations read as:

20 $$\begin{aligned} -\frac{\text {d} \ln q}{\text {d} t}= & {} 1+p\frac{q-u}{q}\nonumber \\ -\frac{1}{2}\frac{\text {d} \ln u}{\text {d} t}= & {} 1+p\frac{z-1}{z}\frac{q-u}{q}. \end{aligned}$$

Combining the two equations:

21 $$\begin{aligned} \frac{z-1}{z}\frac{\text {d} \ln q}{\text {d} t}-\frac{1}{2}\frac{\text {d} \ln u}{\text {d} t}=\frac{1}{z}, \end{aligned}$$

from which we obtain the relation:

22 $$\begin{aligned} u(t)=q^{2\tfrac{z-1}{z}}e^{-\tfrac{2}{z}t}, \end{aligned}$$

with

23 $$\begin{aligned} -\frac{\text {d} \ln q}{\text {d} t}=1+p-pq^{\tfrac{z-2}{z}}e^{-\tfrac{2}{z}t}. \end{aligned}$$

The solution of (23) for $z>2$ is given by:

24 $$\begin{aligned} q(t)=\exp \left( \tfrac{2t}{z-2}\right) \left( \frac{p \tfrac{z-2}{z}+\exp \left[ t\left( 1+p\tfrac{z-2}{z}\right) \right] }{p \tfrac{z-2}{z} +1}\right) ^{\tfrac{z}{2-z}}. \end{aligned}$$

while for $z=2$ it is $q(t)=\exp [-(1+p)t+p(1-e^{-t})]$.

Then the basic correlation functions are given by: $x(t)=1-q(t)$ and $x_2(t)=1-2q(t)+u(t)$.

*The photochemical utilization (absorption) of the exciton* can be realised after $k=1,2,\ldots , n$ steps, provided the RC in the *k*-th step is open, but the RCs are closed in the previous $k-1$ steps. The sum of these contributions is given by:

25 $$\begin{aligned} A=\, & {} \langle 1-\sigma \rangle +p\langle \sigma_{i_1}(1-\sigma_{i_2})\rangle + p^2\langle \sigma_{i_1}\sigma_{i_2}(1-\sigma_{i_3})\rangle \nonumber \\&+\,\cdots +p^{n-1}\langle \sigma_{i_1}\sigma_{i_2} \ldots \sigma_{i_{n-1}}(1-\sigma_{i_n})\rangle =\nonumber \\= \,& {} \sum_{k=1}^{n}p^{k-1}(G_{k-1}-G_k) , \end{aligned}$$

where $G_0=1$, $G_1=x$ and $A+\varphi =1$. Then the absorption cross section is defined as:

26 $$\begin{aligned} \sigma_A=\,\frac{A}{P_{\circ }}=\frac{1-\varphi }{1-x}, \end{aligned}$$

which in the Joliot theory is given by $\sigma_A={1}/({1-px})$.

*The average number of steps*, $\langle n \rangle$, is given by:

27 $$\begin{aligned} \langle n \rangle=\, & {} (1-p)\sum_{k=1}^{n-1}kp^{k-1}G_k+np^n G_n\nonumber \\&+\,\sum_{k=1}^{n}kp^{k-1}(G_{k-1}-G_k)\nonumber \\= \,& {} \sum_{k=0}^{n-1}p^{k}G_k=1+\frac{p}{1-p}(\varphi -p^{n-1}G_n). \end{aligned}$$

Here in the first and in the second line the contributions from the steps ending with fluorescence and with absorption, respectively, are presented. In the Joliot theory it is given by $\langle n \rangle ={1}/({1-px})$, which is just the absorption cross section.

## References

[1] Mirkovic, T., Ostroumov, E. E., Anna, J. M., van Grondelle, R., Govindjee & Scholes, G. D. Light absorption and energy transfer in the antenna complexes of photosynthetic organisms. *Chem. Rev.* **117**(2), 249-293 (2016). 10.1021/acs.chemrev.6b00002 27428615

[2] Maróti, P. & Govindjee. Energy conversion in photosynthetic bacteria. *Photosynth. Res.* **127**(2), 257–271 (2016). 10.1007/s11120-015-0175-0 26216496

[3] Maróti P (2019). Chemical rescue of $$\text{H}^+$$ delivery in proton transfer mutants of reaction center of photosynthetic bacteria. Biochim. Biophys. Acta Bioenerg..

[4] Maróti P (2019). Thermodynamic view of proton activated electron transfer in the reaction center of photosynthetic bacteria. J. Phys. Chem. B.

[5] Franck J, Teller E (1938). Migration and photochemical action of excitation energy in crystals. J. Chem. Phys..

[6] Niederman RA (2016). Development and dynamics of the photosynthetic apparatus in purple phototrophic bacteria. Biochim. Biophys. Acta.

[7] Vredenberg WJ, Duysens LNM (1963). Transfer and trapping of excitation energy from bacteriochlorophyll to a reaction center during bacterial photosynthesis. Nature.

[8] Joliot P, Bennoun P, Joliot A (1973). New evidence supporting energy transfer between photosynthetic units. Biochim. Biophys. Acta.

[9] Paillotin G (1976). Capture frequency of excitations and energy transfer between photosynthetic units in the photosystem II. J. Theor. Biol..

[10] Bennett DIG, Fleming GR, Amarnath K (2018). Energy-dependent quenching adjusts the excitation diffusion length to regulate photosynthetic light harvesting. PNAS.

[11] Lavergne J, Trissl HW (1995). Theory of fluorescence induction in photosystem-II–derivation of analytical expressions in a model including exciton-radical-pair equilibrium and restricted energy-transfer between photosynthetic units. Biophys. J..

[12] de Rivoyre M, Ginet N, Bouyer P, Lavergne J (2010). Excitation transfer connectivity in different purple bacteria: a theoretical and experimental study. Biochim. Biophys. Acta.

[13] Trissl HW (1996). Antenna organization in purple bacteria investigated by means of fluorescence induction curves. Photosynth. Res..

[14] Den Hollander WTF, Bakker JGC, van Grondelle R (1983). Trapping, loss and annihilation of excitations in a photosynthetic system. I. Theoretical aspects. Biochim. Biophys. Acta.

[15] Fassioli F, Olaya-Castro A, Scheuring S, Sturgis JN, Johnson NF (2009). Energy transfer in light-adapted photosynthetic membranes: from active to saturated photosynthesis. Biophys. J..

[16] Amarnath K, Bennett DIG, Schneider AR, Fleming GR (2016). Multiscale model of light harvesting by photosystem II in plants. PNAS.

[17] Chmeliov J, Trinkunas G, van Amerongen H (2016). Excitation migration in fluctuating light-harvesting antenna systems. Photosynth. Res..

[18] Singharoy A, Maffeo C, Delgado-Magnero KH, Swainsbury DJK, Sener M, Kleinekathöfer U (2019). Atoms to phenotypes: molecular design principles of cellular energy metabolism. Cell.

[19] Sebban P, Barbet JC (1985). Simulation of the energy migration in the antenna of purple bacteria by using the Monte Carlo method. Photobiochem. Photobiophys..

[20] Asztalos E, Sipka G, Maróti P (2015). Fluorescence relaxation in intact cells of photosynthetic bacteria: donor and acceptor side limitations of reopening of the reaction center. Photosynth. Res..

[21] Maróti P, Pessarakli M (2016). Induction and relaxation of bacteriochlorophyll fluorescence in photosynthetic bacteria. Handbook of Photosynthesis.

[22] Küpper H, Benedikty Z, Morina F, Andresen E, Mishra A, Trtílek M (2019). Analysis of OJIP cChlorophyll fluorescence kinetics and $$\text{ Q}_{{\rm A}}$$ reoxidation kinetics by direct fast imaging. Plant Physiol..

[23] Niederman RA (2013). Membrane development in purple photosynthetic bacteria in response to alterations in light intensity and oxygen tension. Photosynth. Res..

[24] Feng X, Deng Y, Blöte HWJ (2008). Percolation transitions in two dimensions. Phys. Rev. E.

[25] Jacobsen JL (2014). High-precision percolation thresholds and Potts-model critical manifolds from graph polynomials. J. Phys. A Math. Theor..

[26] Wraight CA, Clayton RK (1974). The absolute quantum efficiency of bacteriochlorophyll photooxidation in reaction centers of *Rhodopseudomonas sphaeroides*. Biochim. Biophys. Acta.

[27] Timpmann K, Zhang FG, Freiberg A, Sundstrom V (1993). Detrapping of excitation energy from the reaction centre in the photosynthetic purple bacterium *Rhodospirillum rubrum*. Biochim. Biophys. Acta..

[28] Scheuring S, Levy D, Rigaud JL (2005). Watching the components of photosynthetic bacterial membranes and their in situ organisation by atomic force microscopy. Biochim. Biophys. Acta.

[29] Liu LN, Scheuring S (2013). Investigation of photosynthetic membrane structure using atomic force microscopy. Trend Plant Sci..

[30] Xin Y, Shi Y, Niu T, Wang Q, Niu W, Huang X, Ding W, Yang L, Blankenship RE, Xu X, Sun F (2018). Cryo–EM structure of the RC-LH core complex from an early branching photosynthetic prokaryote. Nat. Commun..

[31] Kramer T, Rodriguez M (2017). Two-dimensional electronic spectra of the photosynthetic apparatus of green sulfur bacteria. Sci. Rep..

[32] Panitchayangkoon G, Hayes D, Fransted KA, Caram JR, Harel E, Wen J, Blankenship RE, Engel GS (2010). Long-lived quantum coherence in photosynthetic complexes at physiological temperature. PNAS.

[33] Ishizaki A, Fleming GR (2012). Quantum coherence in photosynthetic light harvesting. Annu. Rev. Condens. Matter Phys..

[34] Ball P (2018). Is photosynthesis quantum-ish?. Phys. World.

[35] Comayras F, Jungas C, Lavergne J (2005). Functional consequences of the organization of the photosynthetic apparatus in *Rhodobacter sphaeroides*. I. Quinone domains and excitation transfer in chromatophores and reaction center-antenna complexes. J. Biol. Chem..

[36] Olsen JD, Martin EC, Hunter CN (2017). The PufX quinone channel enables the light-harvesting 1 antenna to bind more carotenoids for light collection and photoprotection. FEBS Lett..

[37] Sipka G, Maróti P (2018). Photoprotection in intact cells of photosynthetic bacteria: quenching of bacteriochlorophyll fluorescence by carotenoid triplets. Photosynth. Res..

[38] Sipka G, Kis M, Smart JL, Maróti P (2018). Fluorescence induction of photosynthetic bacteria. Photosynthetica.

[39] Chi SC, Mothersole DJ, Dilbeck P (2015). Assembly of functional photosystem complexes in *Rhodobacter sphaeroides* incorporating carotenoids from the spirilloxanthin pathway. Biochim. Biophys. Acta.

[40] Sambrook J, Fritsch EF, Maniatis T (1989). Molecular Cloning: A Laboratory Manual.

[41] Siström WR (1977). Transfer of chromosomal genes mediated by plasmid r68.45 in *Rhodopseudomonas sphaeroides*. J. Bacteriol..

[42] Donohue TJ, Kaplan S (1991). Genetic techniques in rhodospirillaceae. Methods Enzymol..

[43] Kis M, Asztalos E, Sipka G, Maróti P (2014). Assembly of photosynthetic apparatus in *Rhodobacter sphaeroides* as revealed by functional assessments at different growth phases and in synchronized and greening cells. Photosynth. Res..

[44] Bína D, Litvín R, Vácha F (2010). Absorbance changes accompanying the fast fluorescence induction in the purple bacterium *Rhodobacter sphaeroides*. Photosynth. Res..

[45] Kocsis P, Asztalos E, Gingl Z, Maróti P (2010). Kinetic bacteriochlorophyll fluorometer. Photosynth. Res..

